# *In vivo *glioblastoma growth is reduced by apyrase activity in a rat glioma model

**DOI:** 10.1186/1471-2407-6-226

**Published:** 2006-09-23

**Authors:** Fernanda B Morrone, Diogo L Oliveira, Patrícia Gamermann, Joseli Stella, Suzana Wofchuk, Márcia R Wink, Luise Meurer, Maria Isabel A Edelweiss, Guido Lenz, Ana Maria O Battastini

**Affiliations:** 1 Departamento de Bioquímica, ICBS, UFRGS, Porto Alegre, RS, Brazil; 2 Departamento de Biofísica IB, UFRGS, Porto Alegre, RS, Brazil; 3 Departamento de Patologia, HCPA, UFRGS, Porto Alegre, RS, Brazil; 4 Faculdade de Farmácia, PUCRS, Porto Alegre, RS, Brazil

## Abstract

**Background:**

ATP is an important signalling molecule in the peripheral and central nervous system. Both glioma growth and tumor resection induces cell death, thus liberating nucleotides to the extracellular medium. Nucleotides are hydrolyzed very slowly by gliomas when compared with astrocytes and induce neuronal cell death and glioma proliferation. The objective of the present study was to test the involvement of extracellular ATP in glioblastoma growth in a rat glioma model.

**Methods:**

To deplete the extracellular ATP, the enzyme apyrase was tested on the treatment of gliomas implanted in the rats CNS. One million glioma C6 cells in 3 microliters of DMEM/FCS were injected in the right striata of male Wistar rats, 250–270 g. After 20 days, the rats were decapitated and the brain sectioning and stained with hematoxylin and eosine. We performed immunohistochemical experiments with Ki67, CD31 and VEGF. Total RNA was isolated from cultured glioma C6 cells and the cDNA was analyzed by Real Time-PCR with primers for the NTPDase family.

**Results:**

C6 glioma cells effectively have a low expression of all NTPDases investigated, in comparison with normal astrocytes. The implanted glioma co-injected with apyrase had a significant reduction in the tumor size (p < 0.05) when compared with the rats injected only with gliomas or with gliomas plus inactivated apyrase. According to the pathological analysis, the malignant gliomas induced by C6 injection and co-injected with apyrase presented a significant reduction in the mitotic index and other histological characteristics that indicate a less invasive/proliferative tumor. Reduction of proliferation induced by apyrase co-injection was confirmed by counting the percentage of Ki67 positive glioma cell nuclei. According to counts with CD31, vessel density and neoformation was higher in the C6 group 20 days after implantation. Confirming this observation, rats treated with apyrase presented less VEGF staining in comparison to the control group.

**Conclusion:**

These results indicate that the participation of extracellular ATP and the ecto-nucleotidases may be associated with the development of this type of brain tumor in an *in vivo *glioma model.

## Background

Gliomas are a type of primary brain tumor that display an extensive invasive behavior, but do not metastasize. The growth and invasiveness into the surrounding normal brain tissue makes gliomas a major challenge for clinical intervention [1-3]. Therefore, the elucidation of the mechanisms involved in tumor growth, invasion and angiogenesis could aid in the treatment of these tumors.

The invasive cellular behavior of malignant gliomas is determined by receptor mediated cell-substratum contacts and cell-cell interaction, as well as cellular locomotion [4]. Clues to the invasion process have been ascertained through clarification of the key roles played by the extracellular matrix (ECM), cell-adhesion molecules and matrix degrading proteases [5].

Among other elements, vascular endothelial growth factor (VEGF) is one of the most prominent angiogenic growth factors. In fact, it has been shown that VEGF is secreted by the C6 rat glioma cell line [6]. This growth factor is produced by almost all solid tumors and its receptors are highly expressed on vascular endothelial cells and, predominantly, in vessels in the proximity of the tumor [7]. Moreover, some other systems may be involved in the growth and invasion of the gliomas. Glutamate secreting glioma cells demonstrate a growth advantage and glioma cells release excitotoxic concentrations of glutamate, thus killing neurons close to the tumor border and opening space for tumor growth [8-10].

Another important signalling molecule that may be involved in glioma development is ATP [11,12]. In glioma C6 cells, ATP can bind and activate P2Y2, whereas its degradation product, ADP, activate P2Y1 and P2Y12 receptors [13-16].

In previous studies, we showed the involvement of the purinergic system in glioma proliferation in different cell types [17] and that glioma cells present a clear resistance to death induced by cytotoxic concentrations of ATP when compared with normal brain tissue [18]. Taken together with its inability to metabolize extracellular ATP [19], the purinergic system may form part of a novel and important mechanism associated with the malignance of these tumors.

ATP may be liberated into the extracellular space by the excitotoxic death of the normal host cells and by the injury caused by tumor resection, which is the mainstay of initial therapy for gliomas [20]. We hypothesize that ATP liberated under these conditions may, besides inducing proliferation of the glioma itself, induce the cell death of normal tissue and, consequently, increase the interstitial space around the malignant glioma.

To test this hypothesis, we examined the effect of co-injection of apyrase, an ATP depleting enzyme, in a C6 rat glioma experimental model, which has been extensively used to test antitumoral interventions.

## Methods

### Cell culture

The C6 rat glioma cell line was obtained from the American Type Culture Collection (Rockville, Maryland, U.S.A.). Cells were grown in culture flasks in Dulbecco's Modified Eagle's medium (DMEM) with 5% fetal calf serum (FCS) (Cultilab, Brazil).

### Glioma implantation

Rat C6 glioma cells at around 70% confluency were trypsinized, washed once in DMEM/5% FCS, spun down and ressuspended in the same medium. A total of one million cells in a volume of 3 μl were injected at a depth of 6.0 mm in the right striata (coordinates with regard to bregma: 0.5 mm posterior and 3.0 mm lateral) of anesthetized male Wistar rats, 250–270 g [9]. After 20 days, the rats were decapitated and the entire brain was removed, sectioned and fixed with 10% paraformaldehyde.

All the procedures were approved by the Ethical Committee of the Hospital de Clinicas de Porto Alegre.

### Treatment with temozolomide

Ten days post-implantation, the rats were treated with 5 mg/Kg/day (i.p.) of temozolomide or DMSO (control group of temozolomide) during 5 consecutive days. The drug was dissolved in DMSO at a final concentration of 10% [21].

### Co-injection of apyrase with C6 cells

Just before the injection, an apyrase (VII grade) (Sigma Chemical Co., St. Louis, MO, USA) solution containing 2 U/ml was prepared in a C6 cell suspension in DMEM/FCS. Cell viability was evaluated by trypan blue exclusion and MTT assays. This preparation, containing C6 cells and apyrase, was injected in 3 μl. An additional pulse of 3 μl DMEM/FCS with apyrase was given within 1 min of the first injection. As a control group, apyrase was denatured by boiling for five minutes and was then injected together with a C6 cell suspension using the same protocol described above.

The enzyme activity of the injected apyrase was controlled by measuring the hydrolysis of ATP and ADP in an incubation medium containing 50 mM Tris.HCl, pH 7.5 and 1.5 mM CaCl_2 _and ATP or ADP as substrates to a final concentration of 1 mM, at 37°C. The reaction was stopped by the addition of 0.2 ml of 10% TCA. The amount of inorganic phosphate liberated was measured by the malachite green method [22].

### Pathological analysis and tumor volume quantification

At least three hematoxylin and eosin (H&E) sections (4 μm thick, paraffin embedded) of each tumor were analyzed by two independent pathologists, blinded for the experimental data. For tumor size quantification, images were captured using a digital camera connected to the microscope and analyzed using Image Tool Software™. The total volume (mm^3^) of the tumor was computed by summing the segmented areas and by the multiplication of the slice resolution.

### Immunohistochemical staining

Paraffin embedded, 5-mm formalin fixed tissue sections were mounted on microscope slides. Tissue sections were then dried overnight at 60°C, dewaxed in xylene and rehydrated with distilled water. Endogenous peroxidase was inhibited by 1% H_2_O_2 _in methanol for 10 min. Incubation with the following antibodies was performed overnight at room temperature: anti-Ki67 (1:20) (Dako, USA) and anti-VEGF (1:400) (Dako, USA), followed by incubation with secondary antibody and Streptavidin-Avidin-Biotin (Kit Lsab, Dako, USA). The peroxidase reaction was performed using 3,3'diaminobenzidine tetrahydrochloride (DAB), according to the manufacturer's specifications. Finally, sections were counterstained with Harris haematoxylin. Glioma cell proliferation was assessed by counting the percentage of Ki67 positive glioma cell nuclei in five independent high-magnification (×200) fields per animal. Sections of rat spleen were used as positive controls.

To evaluate microvessel density, we used anti-CD31 mAb (BD PharMingen, USA). The glioma sample specimens were snap frozen in liquid nitrogen and cut into 4 μm sections. Frozen tissue was fixed in cold acetone (-20°C). Subsequently, serial sections were stained with anti-CD31 mAb (1:30). The reaction was developed as described above. The number of positive vessels/microscopic field (×200) was counted. All samples were read blindly by two independent readers, and the mean of their scores is presented.

### Real time PCR analysis

Total RNA from cortical rat astrocytes or C6 glioma cells was isolated from confluent cultures with RNA Mini Kit (Qiagen) in accordance with the manufacturer's instructions. The cDNA species were synthesized with SuperScript II (Life Technologies) from 5 μg of total RNA in a total volume of 20 μl with both oligo (dT) primer and random hexamers in accordance with the manufacturer's instructions. SYRB Green I-based real-time PCR was carried out using the MJ Research DNA Engine OpticonTM Continuous Fluorescence Detection System (MJ Research Inc., Walthan, MA), as described previously [23]. All PCR mixtures contained: PCR buffer (final concentration: 10 mM Tris-HCl (pH 9.0), 50 mM KCl, 2 mM MgCl_2_, and 0.1% Triton X-100), 250 μM deoxy-NTP (Roche), 0.5 μM of each PCR primer, 0.5× SYBR Green I (Molecular Probes), 5% DMSO, and 1 U taq DNA polymerase (Promega, Madison, WI) with 2 μl cDNA in a 25 μl final volume reaction mix. The samples were loaded into wells of Low Profile 96-well microplates. After an initial denaturation step for 1 min at 94°C, conditions for cycling were 35 cycles of 30 sec at 94°C, 30 sec at 56°C, 1 min at 72°C. The fluorescence signal was measured right after incubation for 5 sec at 79°C following the extension step, which eliminates possible primer dimer detection. At the end of the PCR cycles, a melting curve was generated to identify specificity of the PCR product. For each run, serial dilutions of human GAPDH plasmids were used as standards for quantitative measurement of the amount of amplified DNA. In addition, for normalization of each sample, hGAPDH primers were used to measure the amount of hGAPDH cDNA. All samples were run in triplicate and the data were presented as a ratio of enzymes/GAPDH. The set of primers used for NTPDase1 (CD39), NTPDase2 (CD39L1), NTPDase3 (CD39L3), NTPDase5 (CD39L4) and NTPDase6 (CD39L2) were as described in Vollmayer et al. (2001) [24]. Oligonucleotides were obtained from Invitrogen. Negative controls were performed with water as template and positive controls were plasmids with cDNA sequences for mouse NTPDase1, rat NTPDase2, and human NTPDase3, NTPDase5 and NTPDase6.

### Statistical analysis

Data were analyzed by one-way analysis of variance - ANOVA, followed by Tukey-Kramer test. *P *values < 0.05 were taken to indicate statistical significance and means ± SEM are presented.

## Results

**Figure 1:**
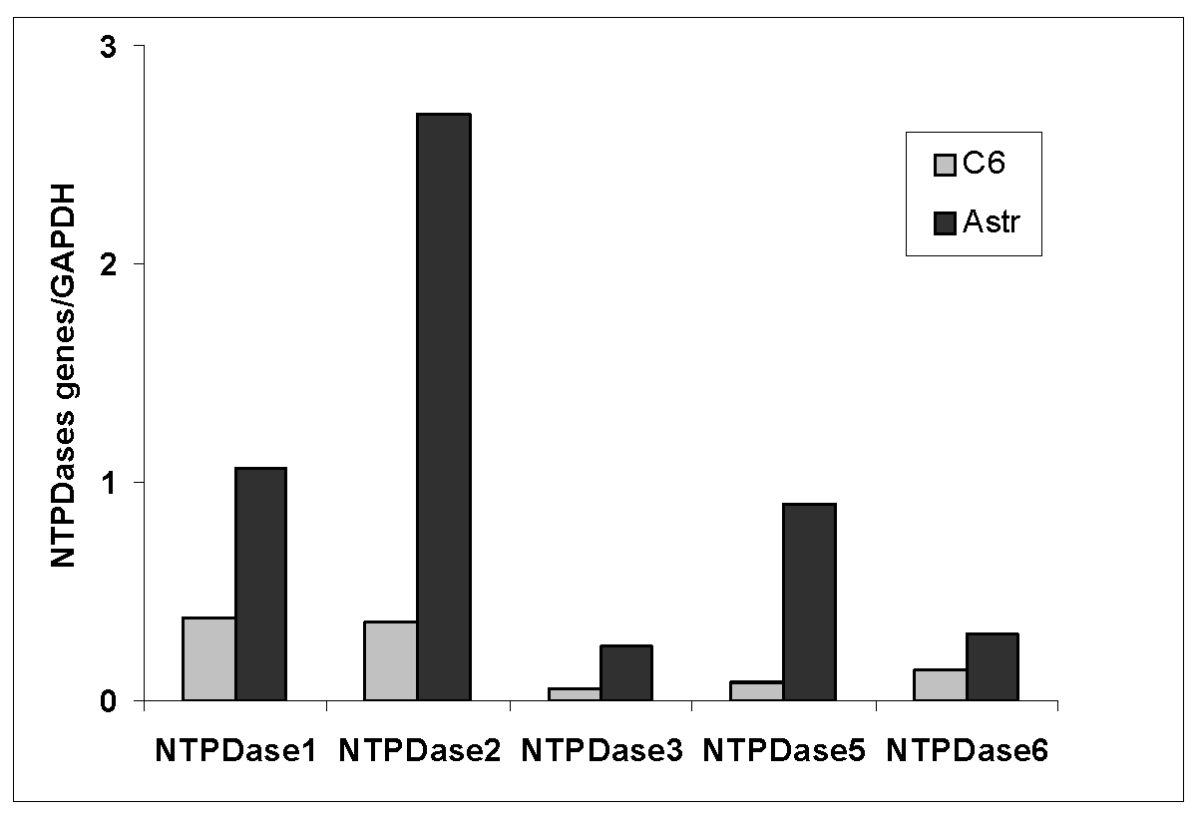
Comparison of NTPDase family expression in rat cortical astrocytes and rat C6 gliomas by quantitative real-time RT-PCR analysis. The expression of NTPDase members in astrocytes and C6 gliomas was quantitatively analyzed by real-time PCR as described in material and methods. C6: rat glioma cell line. Astr: cortical rat astrocytes. The results are presented as a ratio of cDNA enzymes/GAPDH.

In the present study we tested the effect of the apyrase enzyme on the growth of glioma cells *in vivo*. We have previously shown that C6 gliomas, a cell line that was originated from N-nitrosomethylurea-treated rats, exhibited almost undetectable ATP and ADP-degrading activity; however, our results did not allow the identification of which NTPDase family members are expressed by these cells [19]. To investigate which of the ecto-NTPDases are present in the C6 glioma cell line, total RNA was isolated from cultured glioma cells and the cDNA was analyzed by Real Time-PCR with primers for the NTPDase family. Figure 1 shows that C6 cells effectively have a low expression of all NTPDases investigated, in comparison with normal astrocytes. We, therefore, chose the purified apyrase, which hydrolyzes ATP and ADP at a similar rate to test our hypothesis of the ATP participation in the process of glioma invasion in host brain.

The implanted tumors have characteristics that are closer to those of human glioblastomas with the C6 cells growing in the intracerebral, intraventricular and intraparenchymal spaces (data not shown). Haematoxylin and eosin examination showed that the implanted tumor has a high mitotic index, nuclear pleomorphism, foci of tumor necrosis, intratumoral hemorrhage and parenchymal invasion (Table 1). In some cases, pathological analysis identified pallisading cells delineating the foci of necrosis and lymphocytic infiltration, with the occasional formation of edema fluid and neovascularization, which are characteristics of glioblastoma multiforme in humans (Table 1) (Fig. 3a and 3c).

**Figure 2:**
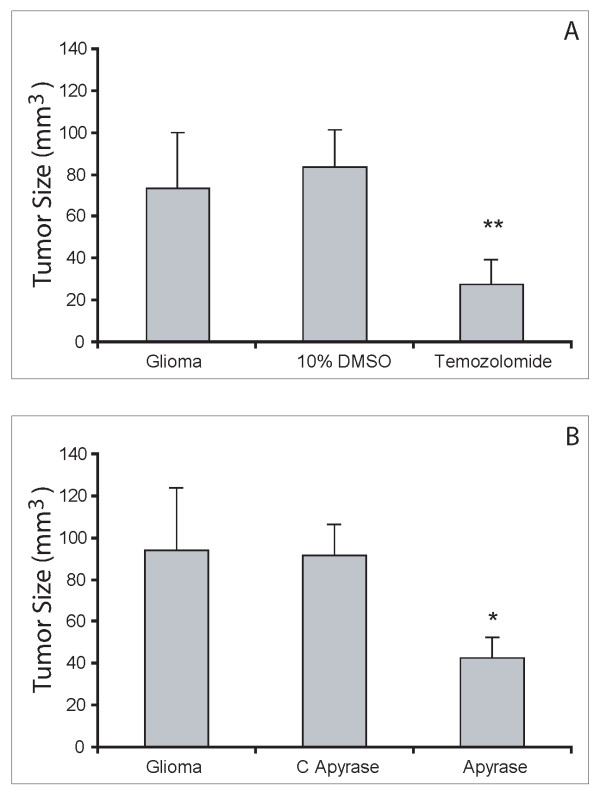
Tumor size of implanted gliomas. Tumor size was measured 20 d after implantation of C6 cells in the different groups. **(A) **Rats were treated with 10% DMSO (vehicle control) or temozolomide in 10% DMSO (n = 5). **(B) **C6 cells were co-injected with: denaturated apyrase (C apyrase) or 2 U of apyrase (apyrase group) (n = 6). Data are means ± SEM. **p < 0.01; *p < 0.05 for comparison versus control, as determined by ANOVA, followed by Tukey-Krammer test.

**Figure 3:**
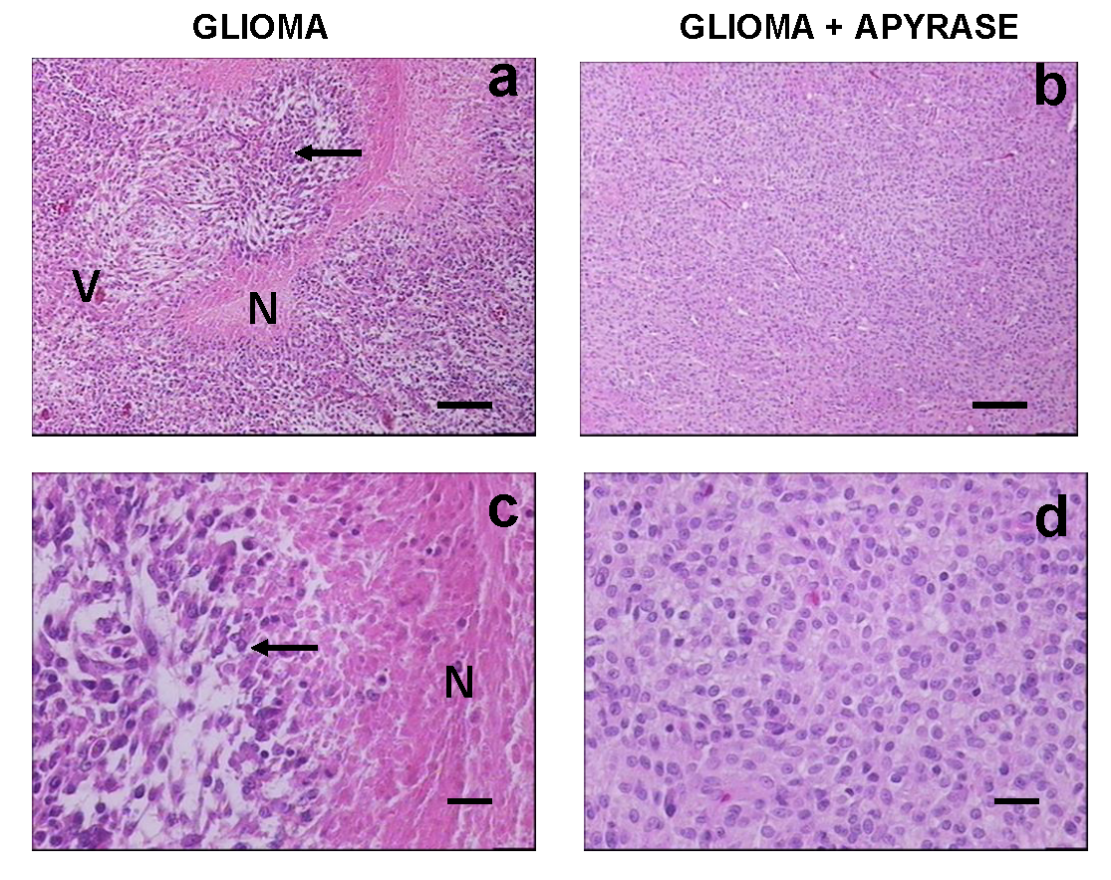
The sections of implanted rat glioma were stained with haematoxylin and eosin (H&E). Histological characteristics that define glioblastoma multiform as seen in rats implanted with gliomas (a,c) and in rats co-injected with apyrase (b,d). Necrosis (N) and microvascular proliferation (V), giant cell formation and nuclear pleomorphism (arrow). Scale bars = 100 μm (a,b); 20 μm (c,d).

In order to analyze whether the tumors implanted into host brain were responsive to drugs that are clinically used to treat gliomas and to validate our model, the rats were treated with the alkylating agent, temozolomide [25]. In fact, tumor size analysis showed that the rats treated with temozolomide presented a significant reduction in tumor size when compared with the control groups (Fig. 2a). In addition, rats treated with temozolomide survive significantly longer than their respective controls, demonstrating a relative benefit of 100% in the rats' survival, that means, at the time that 100% of control animals died all animals from the treated group were still alive (data not shown).

Confirming our purinergic hypothesis of glioma growth, the results presented in Figure 2b show that the rats with implanted glioma co-injected with apyrase had a significant reduction in the tumor size (p < 0.05) in comparison with the rats injected only with gliomas or with gliomas plus inactivated apyrase, indicating that it is not the presence of the polypeptide, but the enzymatic activity that is responsible for the effect of apyrase. The effect of apyrase (2 U apyrase/10^6 ^cells) on C6 cell proliferation in culture showed that the enzyme was not toxic to the cells but instead, induced an increase by around 40% of the cell number after 24 h of treatment. Additionally, the C6 cells were detached from the surface handled exactly as for the injection (with or without apyrase) and were subsequently re-plated in DMEM/FCS for 24 and 48 h. Cell viability was analyzed by trypan blue exclusion and MTT assays immediately after the cell preparation for injection and after 24 and 48 h of culture. The cells presented more than 90% viability when compared to the control cells in all conditions tested, indicating that the effect of apyrase is not probably on the initial viability of the injected cells but rather on the subsequent tumor implantation and growth.

**Figure 4:**
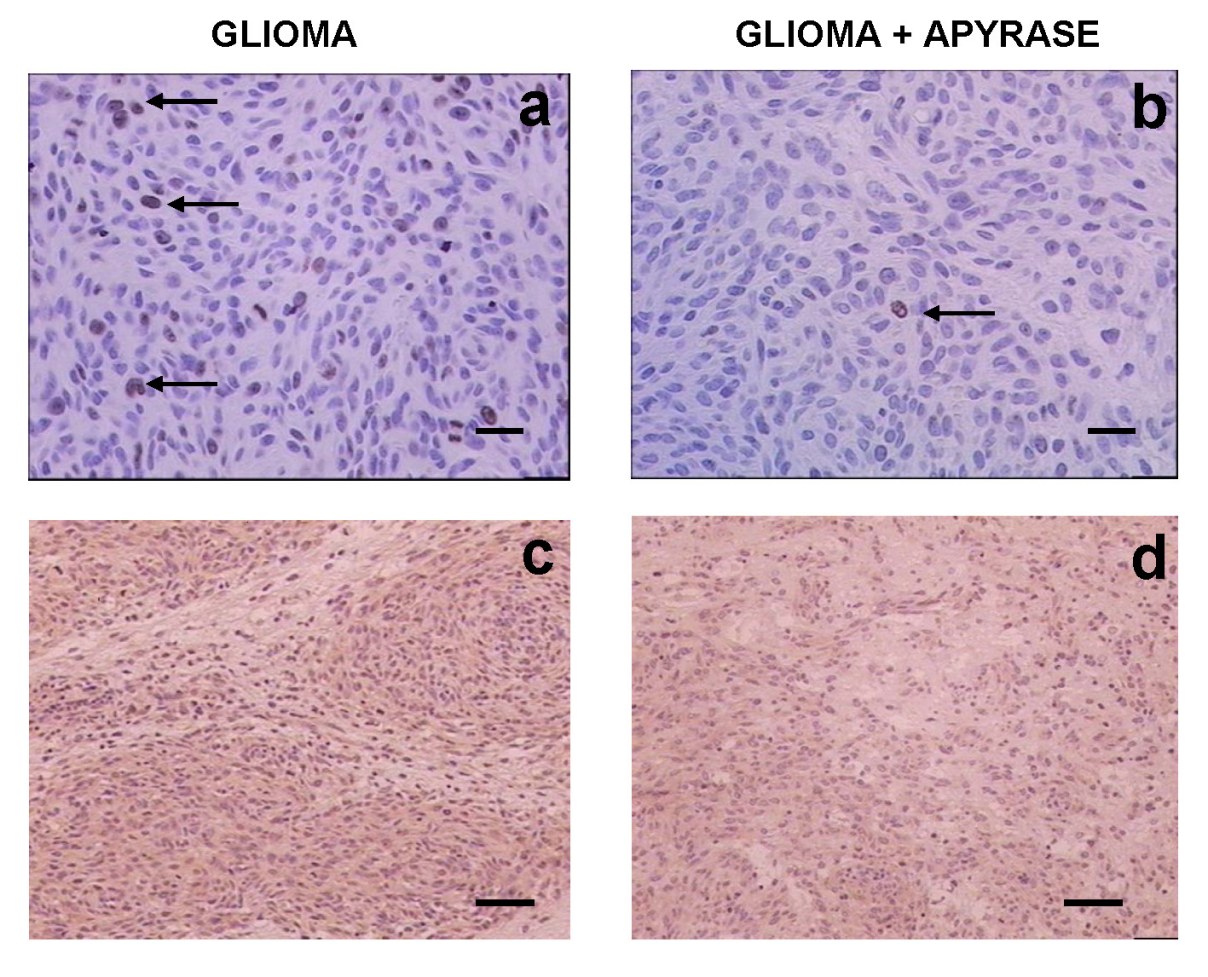
Immunohistochemical stainings of gliomas. Glioma cell proliferation was assessed by immunostaining for Ki67 positive glioma cell nuclei (arrows) in rats implanted with gliomas (a) and in rats co-injected with apyrase (b). The sections were immunostained for VEGF, in rats implanted with gliomas (c) and in rats co-injected with apyrase (d). Scale bars = 20 μm (a,b); 100 μm (c,d).

According to the pathological analysis, the malignant gliomas induced by C6 injection and co-injected with apyrase presented a significant reduction in the mitotic index and other histological characteristics that indicate a less invasive/proliferative tumor (Table 1). Less lymphocytic infiltration and peritumoral edema were observed as well as the absence of pseudopalisading necrosis (Fig. 4b and 4d). Reduction of proliferation induced by apyrase co-injection was confirmed by counting the percentage of Ki67 positive glioma cell nuclei (Table 2) (Fig. 4a and 4b).

Since tumor growth is dependent on the ability to induce angiogenesis, we performed immunohistochemical experiments with CD31. According to counts with CD31, vessel density and neoformation was higher in the C6 group 20 days after implantation, in comparison with the group co-injected with apyrase (Table 2). Confirming this observation, the implanted gliomas with apyrase presented less VEGF staining in comparison to the control group (glioma alone) (Fig. 4c and 4d).

## Discussion

In the present study, we investigated the effect of the co-injection of the enzyme apyrase with implanted C6 gliomas cells. It is well known that *in vitro *assays for growth and invasion only represent isolated aspects of the multi-cascade process of the *in vivo *tumor growth [4]. For this reason, we decided to use an *in vivo *glioma model that, despite its limitations, is useful for the study of growth, angiogenesis and invasion of gliomas [3].

Extracellular ATP could be hydrolyzed by the action of a cascade of enzymes, which includes the family of E-NTPDases: NTPDase1 (CD39), NTPDase2 (CD39L1), NTPDase3 (CD39L3), NTPDase5 (CD39L4) and NTPDase6 (CD39L2) [26] and the more recently described NTPDase7 and NTPDase8 [26-28]. Some of these enzymes have been related with different tumors [29-31] and recent studies demonstrated that NTPDase5 (CD39L4) is a proto-oncogene PCPH, which is involved in tumor growth [32]. According to our results, the C6 cells present a very low expression of the NTPDase1 (CD39), NTPDase2 (CD39L1) and NTPDase3, the three main enzymes involved in the cell surface ATP degradation when compared to astrocytes. Previous studies showed that nucleotides and nucleosides induce proliferation in glioma cell lines [17] while ATP can mediate death in dissociated primary cerebellar granules or striatal neurons and in hippocampal organotypic cultures [33]. The indirect autocrine nature of this process should be noted. The tumor induces, through the death of normal cells, the release of more ATP that stimulates its own multiplication and, as a consequence, the cell death of other cells opening space to be occupied by tumor cells and liberating more ATP to continue the invasive process. Thus we hypothesize that the re-establishment of NTPDase activity may reduce glioma growth.

Purified apyrase is an enzyme widely used to deplete ATP and hydrolyzes ATP and ADP at the same rate as NTPDase1 [34]. Therefore, the apyrase may regulate the extracellular nucleotide concentration and, thus, modulate their biological effects when activated by P2Y and P2X receptors.

Our experiments demonstrate that the co-injection of apyrase significantly diminished the growth of implanted gliomas in rats after twenty days of tumor induction. Considering that the injection of apyrase was done only at the moment of implantation, the effect of ATP depletion is probably at the implantation and initial growth of the glioma. This could be of therapeutic interest, since the application of apyrase in the surgical resection cavity could be helpful in reducing the initial growth of invaded tumor.

In addition to the reduction in the glioma size, pathological analysis demonstrated the lack of some important malignant characteristics, typical of the glioblastomas, in the tumors co-injected with apyrase (Table 1). Among these, of note is the reduction of the mitotic index in the rats treated with apyrase, indicating that ATP has an important role in glioma proliferation *in vivo*. The participation of ADP in this process should not be excluded since this nucleotide can bind to P2 receptors and is potentially hydrolyzed by the injected apyrase.

As mentioned above, angiogenesis is an extremely important process for sustained tumor growth. Studies have demonstrated that C6 and human glioma cell lines secrete VEGF [6,35,36]. Since invasive properties are largely dependent on the presence of new vessels, CD31 was studied and demonstrated a greater staining in the samples from rats implanted with the C6 cells in relation to those co-injected with apyrase (Table 2). This result was confirmed by the stronger VEGF staining in rats implanted with glioma, when compared with the apyrase group (Fig. 4c and 4d). These results clearly indicate that microvascular proliferation is decreased in tumors treated with apyrase in relation to the control group, with a consequent decrease in the blood flow in tumor areas with high cell proliferation. The insufficient vascular supply may become the limiting factor for tumor growth [7].

## Conclusion

In conclusion, the data presented herein clearly show that the presence of an ATP-depleting enzyme at the moment of glioma implantation causes a significant decrease in the growth, angiogenesis and proliferation index. The exact mechanisms to explain these observations remain under evaluation, however, the participation of ATP and the ecto-nucleotidases may be associated with the development of this type of brain tumor in an *in vivo *glioma model.

## Abbreviations

CNS, central nervous system; DAB, 3,3'diaminobenzidine tetrahydrochloride; DMEM, dulbecco's modified eagle's medium; DMSO, dimethyl sulfoxide; ECM, extracellular matrix; FCS, fetal calf serum; H&E, hematoxylin and eosin; VEGF, vascular endothelial growth factor.

## Competing interests

The author(s) declare that they have no competing interests.

## Authors' contributions

FBM performed cell growth, glioma injection, drug treatments, conducted H&E and immunohistochemical analysis of gliomas, was responsible for data analysis and drafting of the manuscript. DLO participated in the glioma injection. PWG performed drug treatments and tumor size measurements. JS performed cell growth. SW participated in the glioma injection and glutamate uptake assay. MRW carried out Real Time PCR studies. LM and MIAE conducted H&E and immunohistochemical analysis of gliomas. GL participated in the study design and drafting of the manuscript. AMOB conceived of the study, participated in its design, coordination and in the draft of the manuscript. All authors read and approved the final manuscript.

## Pre-publication history

The pre-publication history for this paper can be accessed here:



## Figures and Tables

**Table 1:** Characteristics of implanted gliomas.

	**Glioma (n = 9)**	**Glioma + Apyrase (n = 8)**
Coagulative necrosis	5/9	0/8
Intratumoral Hemorrhage	2/9	1/8
Lymphocytic infiltration	8/9	5/8
Peritumoral edema	5/9	1/8
Peripheric pseudopalisading	3/9	0/8
Mitotic index: mitosis/HPF	14.5 ± 4.7	8.4 ± 2.9*

The histological variables (coagulative necrosis, intratumoral hemorrhage, lymphocytic infiltration, peritumoral edema, peripheric pseudopalisading) were regarded as present or absent. Mitosis was counted in ten high power fields (HPF) of the periphery of the lesion, and the average of this counting was used as mitotic index. *P < 0.05, Students *t*-Test.

**Table 2:** Immunohistochemical analysis of Ki67 and CD31 in rats implanted glioma.

	**Glioma**	**Glioma + Apyrase**
Ki67 # (n = 5)	48.7 ± 2.3	11.6 ± 7.9**
CD31◆ (n = 2)	96 ± 22.6	37.5 ± 3.5

# percentage of positive cells. Data are the means ± SEM

◆ Data are the means ± SEM for the number of vessels

**p < 0.01, Students *t*-test.
